# Characterization of sinoatrial automaticity in *Microcebus murinus* to study the effect of aging on cardiac activity and the correlation with longevity

**DOI:** 10.1038/s41598-023-29723-5

**Published:** 2023-02-21

**Authors:** Mattia L. DiFrancesco, Manon Marrot, Eleonora Torre, Pietro Mesirca, Romain Davaze, Corinne Lautier, Pascaline Fontes, Joël Cuoq, Anne Fernandez, Ned Lamb, Fabien Pifferi, Nadine Mestre-Francés, Matteo E. Mangoni, Angelo G. Torrente

**Affiliations:** 1 Institut de Génomique Fonctionnelle, Université de Montpellier, CNRS, INSERM, Montpellier, France; 2 LabEx Ion Channels Science and Therapeutics (ICST), Sophia Antipolis, France; 3 Institute of Human Genetics, CNRS and University of Montpellier, Montpellier, France; 4 MMDN Univ Montpellier, EPHE, INSERM, Montpellier, France; 5 UMR CNRS/MNHN 7179, Mécanismes Adaptatifs et Evolution, Brunoy, France; 6 PSL Research University, Paris, France; 7 Present Address: IRCCS Ospedale Policlinico San Martino, Largo Rosanna Benzi 10, 16132 Genoa, Italy

**Keywords:** Physiology, Zoology, Cardiology

## Abstract

*Microcebus murinus,* or gray mouse lemur (GML), is one of the smallest primates known, with a size in between mice and rats. The small size, genetic proximity to humans and prolonged senescence, make this lemur an emerging model for neurodegenerative diseases. For the same reasons, it could help understand how aging affects cardiac activity. Here, we provide the first characterization of sinoatrial (SAN) pacemaker activity and of the effect of aging on GML heart rate (HR). According to GML size, its heartbeat and intrinsic pacemaker frequencies lie in between those of mice and rats. To sustain this fast automaticity the GML SAN expresses funny and Ca^2+^ currents (*I*_*f*_, *I*_*Ca,L*_ and *I*_*Ca,T*_) at densities similar to that of small rodents. SAN automaticity was also responsive to β-adrenergic and cholinergic pharmacological stimulation, showing a consequent shift in the localization of the origin of pacemaker activity. We found that aging causes decrease of basal HR and atrial remodeling in GML. We also estimated that, over 12 years of a lifetime, GML generates about 3 billion heartbeats, thus, as many as humans and three times more than rodents of equivalent size. In addition, we estimated that the high number of heartbeats per lifetime is a characteristic that distinguishes primates from rodents or other eutherian mammals, independently from body size. Thus, cardiac endurance could contribute to the exceptional longevity of GML and other primates, suggesting that GML’s heart sustains a workload comparable to that of humans in a lifetime. In conclusion, despite the fast HR, GML replicates some of the cardiac deficiencies reported in old people, providing a suitable model to study heart rhythm impairment in aging. Moreover, we estimated that, along with humans and other primates, GML presents a remarkable cardiac longevity, enabling longer life span than other mammals of equivalent size.

## Introduction

Heart rate (HR) and lifetime are correlated to mammals’ body size and are thought to be mutually related^1^. Typically, small mammals have fast HR to warm up their bodies and contrast heat dispersion^2,3^, while large mammals, that better maintain body warmth, have lower HR^4^. Accordingly to the energy expenditure necessary to maintain body warmth, small mammals have shorter lifespan than large mammals, something that has been also related to a maximum of 1 billion heartbeats generated by the heart of most mammals in a lifetime^1,5^. Humans can attain ~ 3 billion heartbeats, making an exception that was supposed to be due to the improved life conditions in modern societies^6^.

**Figure 1:**
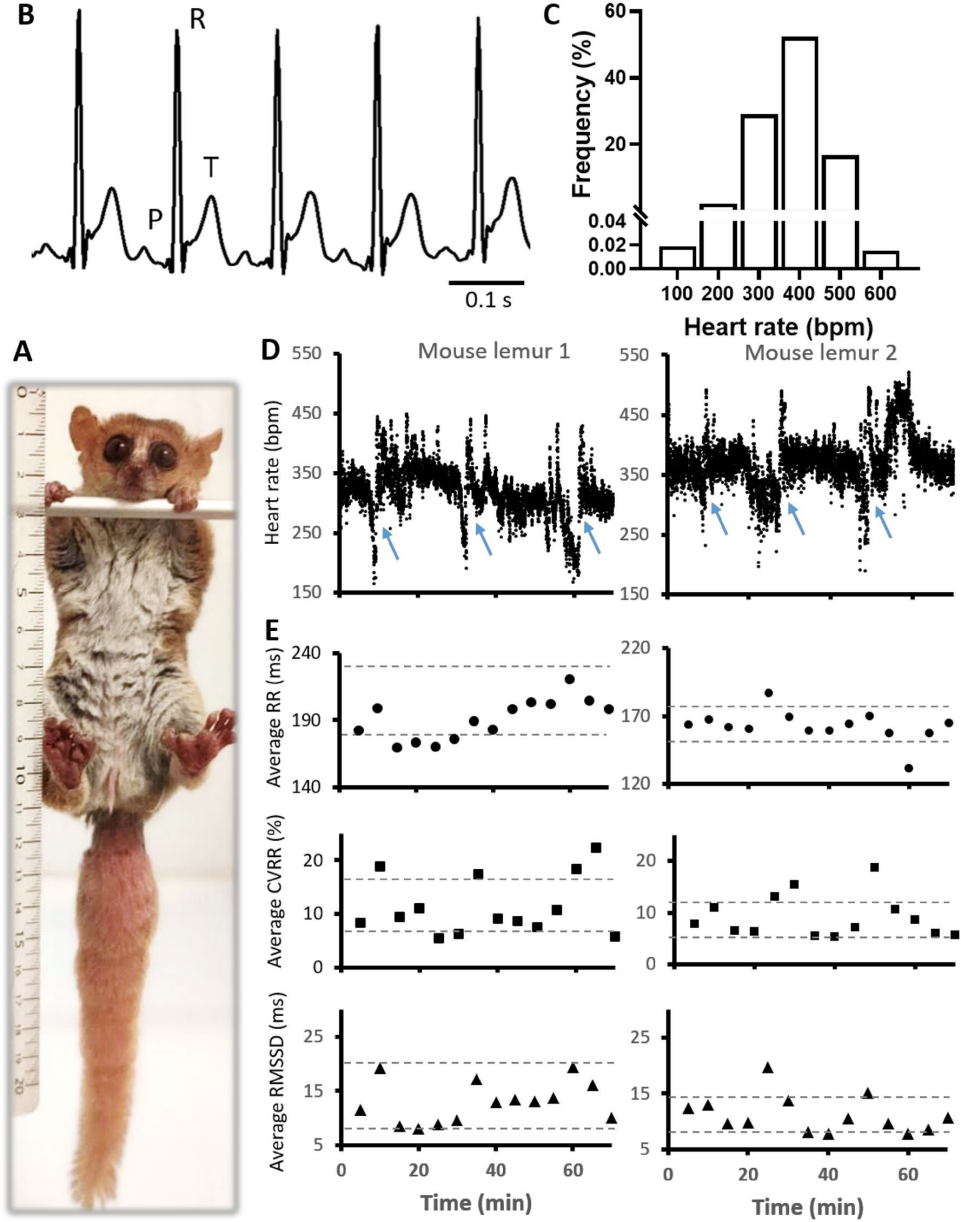
In vivo recording of heart rate (HR) in GML. (**A**) 4 years old female of GML (*Microcebus murinus*). (**B**) Sample traces of ECGs in freely moving GMLs. Note P, R and T waves. (**C**) Distribution of heart rates (HR) measured during 22 continuous hours of ECG recordings in one GML. Bars represent the percentage of binned HR values. Binning width is 100 bpm. (**D**) Example of 70-min recordings in two GMLs showing HR changes over time. Dots represent consecutive R–R intervals. Note cyclical HR decreases to very low frequencies as indicated by blue arrows. (**E**) Plots obtained by averaging every 5 min the interval between successive heartbeats (RR interval), its coefficient of variability (CVRR) and the root mean square of successive differences between heartbeats (RMSSD), for the recordings shown in (**C**). Dashed gray lines indicate the threshold of mean ± SD for each of the parameters reported in this panel.

We investigated HR and cardiac automaticity of *Microcebus murinus*, or gray mouse lemur (GML), in relation to aging, because its lifetime largely overpasses what could be expected for such a small animal. Indeed, with a weight of 60–90 g and a body length of 10–12 cm (Fig. 1A) this lemur have a size in between mice and rats and ranks among the smallest primates known^7^, together with the pygmy marmoset (*Cebeulla pygmaea;* 110–130 g)^8,9^, the pygmy mouse lemur (*Microcebus myoxinus*; 43–55 g)^10^ and the Madame Berthe's mouse lemur (*Microcebus berthae;* 30 g)^11^. Moreover, while GML lifespan is 4–5 years in the wild, it can reach 12 years in captivity, undergoing a prolonged period of senescence like humans, and very different from the short life expectancy of 2–4 years in small rodents.

In humans, aging is a major cause of dysfunction of the sinoatrial node (SAN), the cardiac tissue that works as the natural pacemaker of the heart^12^. SAN dysfunction and associated brady-arrhythmias account for about half of the 500,000 electronic pacemakers implanted in Europe annually^13^, costing 2 billion $ per year^14^.

Given the increased incidence of SAN dysfunctions in our aged societies^15^, it is important to investigate species that could better replicate human physiology to understand the effects of aging on the SAN pacemaker activity. Primates would be the first election, but ethical concerns and the consequent difficulties to obtain living hearts in this order of mammals have limited the studies of their pacemaker mechanism^16–20^.

Current understanding of the fundamental mechanism of cardiac pacemaker activity has been mainly based on studies performed mostly in rabbits and mice^21–23^. These studies showed a functional association between plasma membrane ion channels and intracellular Ca^2+^ dynamics to depolarize SAN myocytes^24,25^ and initiate the heartbeat. Among ion channels of the plasma membrane, hyperpolarization-activated HCN4 channels^23^, L-type Ca_v_1.3^25–27^ and T-type Ca_v_3.1 Ca^2+^ channels^28^, underlying “funny”, L-type and T-type Ca^2+^ currents (*I*_*f*_, *I*_*Ca,L*_ and *I*_*Ca,T*_, respectively), are major mechanisms of diastolic depolarization. In addition, Ca^2+^ dynamics initiated by the release of sarcoplasmic Ca^2+^ from the ryanodine receptors (RyRs) and the generation of an inward current through the Na^+^/Ca^2+^ exchanger (NCX) are another mechanism of pacemaker depolarization^22,24,29^. The generation of pacemaker activity triggered by these depolarizing currents, in combination with the autonomic modulation set the resting HR of different animals.

Although mice have been very versatile models to investigate the cardiac pacemaker mechanism^21,30^, genetic distance must be taken into account when comparing physiology of rodents and humans^31^. Moreover, given the relatively short lifespan of rodents, these animals may present limitations to understand the effect of aging on human cardiac physiology.

The long period of senescence in *Microcebus murinus* justifies the use of this lemur to study how aging affects heart activity, helping to bridge the gap between the cardiac knowledge in rodents and humans. GML can be bred easily in animal facilities, thanks to its small size and easy reproduction. Breeding of the species in captivity may help maintain a viable population for future reintroduction in the wild, to fight the threat of habitat loss. Unlike rodents, GML spontaneously develops age-related neurodegenerative diseases similar to humans^32–36^, which could also affect the heart-brain interplay^37^. Since aging can affect the intrinsic pacemaker activity as well as its autonomic modulation, GML could better capture the neurodegenerative context that modulates aged hearts in humans.

To characterize GML cardiac physiology and understand the consequences of aging we studied HR and pacemaker activity of GML at three different levels: (i) freely moving and anesthetized animals, (ii) isolated hearts and SAN tissues, and (iii) single SAN pacemaker myocytes.

We compared cardiac automaticity of GML with previous findings obtained in rats and mice, rodents whose sizes are comparable to that of GML. Moreover, we compared HR, body mass and life expectancy of GML with humans and other mammals belonging to primates, rodents and other orders. Thanks to these comparisons we estimated that the number of heartbeats per lifetime in GML and other primates is equivalent to what can be attained by humans, and three times higher than other eutherian mammals. Thus, GML represents a better model that could replicate a cardiac workload similar to that of the human heart in aging.

## Results

### Analysis of the heart rate (HR) of freely moving and anesthetized GMLs

**Figure 2:**
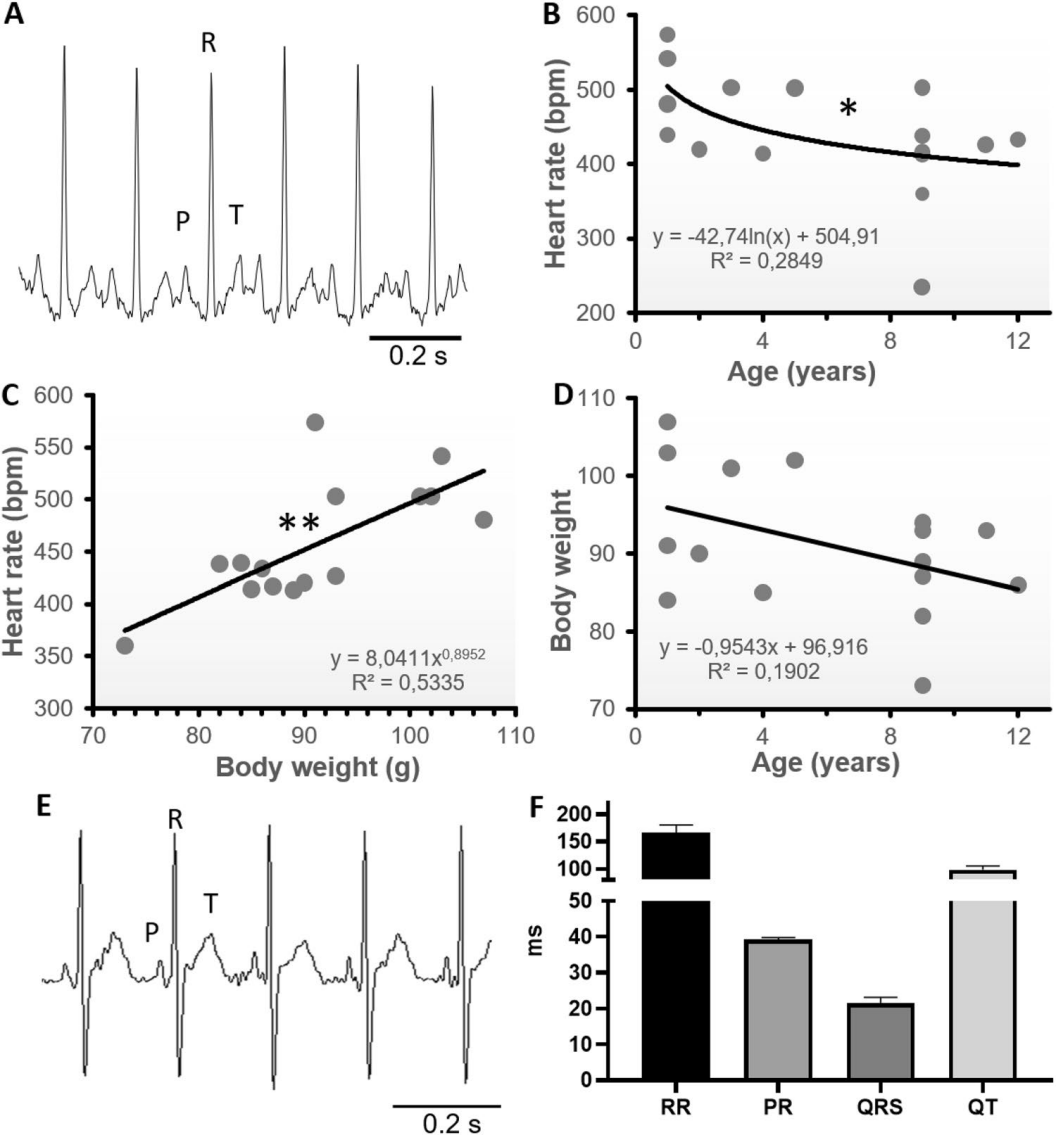
Cardiac activity of GML on surface ECG or during anesthesia, and correlation between HR, age and body weight. (**A**) Electrocardiograms from surface ECGs. Note P, R and T waves. (**B**) Correlation between HR and age in freely moving GML (n = 16); significant by Pearson or Spearman correlation coefficients (*p < 0.05), it respect the normal distribution according to d’Agostino and Pearson test, or Kelmogorov-Smirnov test (*p < 0.05). (**C**) Correlation between HR and body weight in freely moving GML (n = 15); significant by Pearson or Spearman correlation coefficients (**p < 0.01 and *p < 0.05, respectively), it respect the normal distribution according Kelmogorov-Smirnov test (*p < 0.05). One couple of extreme value was excluded from this correlation analysis. (**D**) Correlation between body mass and age (n = 16); Non-significant by Pearson or Spearman correlation coefficients, does not respect the normal distribution. (**E**) Electrocardiograms from anaesthetized GMLs. Note P, R and T waves. (**B**) Interval between successive heartbeats (RR), interval between atrial and ventricular depolarization (PR), length of the ventricular depolarization (QRS) and interval between ventricular depolarization and repolarization (QT) characterizing the ECG waveform of anaesthetized GMLs (n = 5).

We used subcutaneous telemetry transmitters to record electrocardiograms (ECGs) in adult GMLs of 1–5 years old^33,38^ (Fig. 1). This technique allowed us to identify P waves, QRS complex, and T waves, which correspond to atrial depolarization, ventricular depolarization, and ventricular repolarization^39^ (Fig. 1B). The analysis of these ECGs showed averaged basal HR of 427 ± 43 bpm (n = 4; Fig. 2B). Moreover, in 22-h of continuous ECG recording we observed a main range of HR between 200 and 500 bpm (Fig. 1C), with a minimum and maximum HR of 120 and 555 bpm, respectively. These extreme rates are consistent with the maximal and minimal HR (160 and 520 bpm) that we reported in the graphical plots of HR over time (Fig. 1D). In these plots, episodes of gradual HR reduction to ~ 200 bpm appeared to be a recurring pattern. These episodes are too short to correspond to the phase of torpor that could occur in GML when the outside temperature drops^40,41^, but could be determined by short sleeping phases (Fig. 1D). Indeed, the HR variability (HRV) analysis revealed that these episodes of bradycardia have a slow dynamic of several minutes, which differs from normal short-term HRV generated by the parasympathetic regulation^43^. Such HR reductions influence the long-term HRV indexes, like the coefficient of variability of the RR interval, more than the short-term HRV indexes (root mean square of successive differences [RMSSDs]; Fig. 1E). Similar conclusions were supported by the Poincaré analyses of the short-term and long-term HRV indexes (SD1 and SD2, respectively; Fig. S1) that we run on the RR plots.

Next, we used an external device to record surface ECG across GMLs of different ages from 1 to 12 years old (Fig. 2A). According to the literature we considered young or adult, animals whose age range from 1 to 5, and aged the animals whose age range between 6 and 12 years^33,38^. The averaged HR of pooled animals was 444 ± 20 bpm (n = 16), which is similar to what we obtained with implanted animals. This average rate is in between what has been reported in mice^44,45^ and rats^46,47^, consistently with the average size of our pooled GMLs (91 ± 2 g; n = 16).

In addition, we observed that HR in GML significantly decreased with age (Fig. 2B) and increased with body mass (Fig. 2C), while body mass tends to significantly decreased with age (Fig. 2D). Together these three variables indicate that HR can be represented by the linear regression model HR = 280.1–7.7*age (years) + 2.3*weight (g), were age had almost twice more influence in the determination of HR than weight (Standardized β coefficients = − 0.39 and 0.26, for age and weight, respectively).

Finally, we recorded ECGs of the anesthetized GMLs (Fig. 2E,F)*.* The average HR determined under these conditions was 372 ± 27 bpm (n = 5), a value consistent with the decrease in HR induced by anesthesia^48^ that again place the intrinsic HR of GML between those of mice and rats, according to the average GML size^44,47^. As for the implanted animals, under anesthesia we observed P waves, QRS complex and T waves (Fig. 2E,F). In ECGs recorded in freely moving and anaesthetized animals we noticed that differently from small rodents^39^, the T waves were separated from the QRS complexes by short isoelectric ST segments (Figs. 1B and 2E). This segment indicates a longer interval between ventricular depolarization and repolarization in GML’s hearts versus rodents of similar size. In addition, although anesthesia slowed down HR^48^ and perhaps repolarization^49^, the presence of an ST segment both in awaked and anaesthetized GMLs seemed to exclude that the delayed interval between depolarization and repolarization was a consequence of anesthesia.

### Analysis of intrinsic pacemaker activity in isolated hearts and intact atrial preparations

To comply with the “three Rs” of animal welfare (Replacement, Reduction and Refinement), we obtained results on the isolated cardiac tissues of GML only from animals aged 5–12 years that had to be euthanized because of natural aging or other factors interfering with normal life. Post-mortem organ analysis revealed tumours as the main cause of decrease in body weight, activity or other vital signs, with a prevalence of renal and liver pathologies (Table 1). Only a few animals (0.5%) presented evident signs of cardiac hypertrophy after euthanasia.

**Table 1:** Statistic of pathologies reported after animal euthanasia (N GML = 640).

Affected organs	Kidney	Liver	Skin	Lung	Intestine	Abdomen	Ovary	Vesicle	Spleen
% of animals with tumors	8.3%	0.8%	0.8%	0.6%	0.6%	0.6%	0.2%	0.2%	0.2%

	Liquide in the thoracic cage	Liquid in the abdomen	Heart hypertrophy						
% of animal with other pathologies	0.8%	0.6%	0.5%						

**Figure 3:**
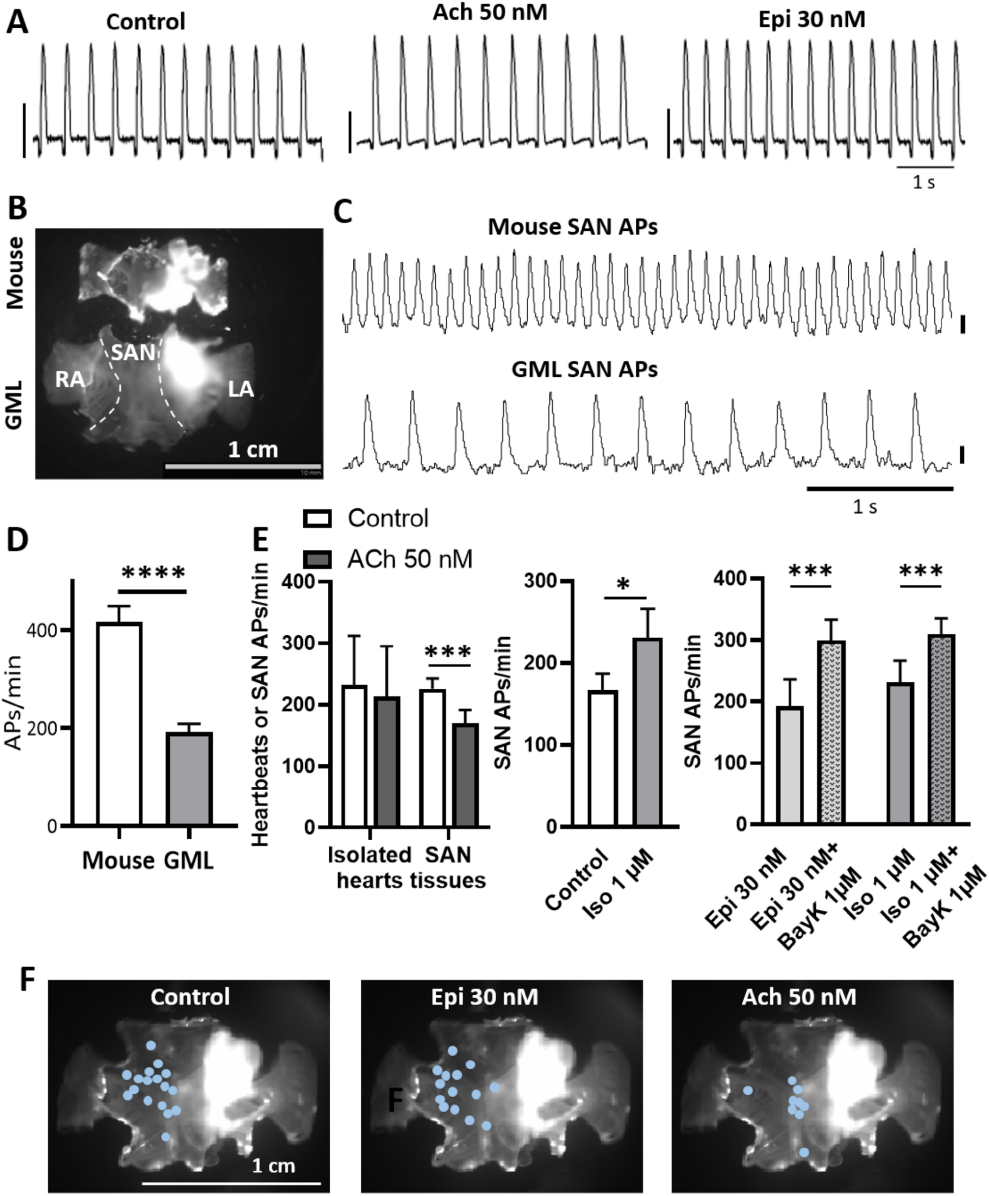
Pacemaker activity of GML isolated hearts and SAN preparations. (**A**) Sample traces of ECGs recorded in isolated hearts from GML, maintained under constant perfusion with a Langendorff system. Traces have been recorded under control conditions (perfusion with Tyrode’s solution), perfusion of acetylcholine (Ach; 50 nM) or epinephrine (Epi; 30 nM). Scale bars correspond to 1 mV. (**B**) Examples of atrial preparations of mice and GML*,* including the SAN, the right and left atria (RA and LA, respectively). (**C**) Example traces of optical action potentials (APs) recorded in the SAN of mice and GMLs. APs amplitude is expressed in arbitrary units of fluorescence (a.u.), scale bars correspond to 1 a.u. (**D**) Rate of APs recorded in mice and GMLs under control condition (n mice = 13, n GMLs = 17). (**E**) Changes in automatic rate (heartbeats or APs/min), before and after Ach or isoprenaline (Iso) superfusion, or during Epi and Iso superfusion and after addition of Bay K 8644 (BayK), in Langendorff perfused hearts (n = 3) or atrial preparation of GML (n = 11 in ACh, n = 10 in Iso, n = 5 in Epi and Epi + BayK and n = 10 in Iso and Iso + BayK). (**E**) Dots indicating leading pacemaker sites in SAN preparations of GML under control, Epi or Ach perfusion. *p < 0.05, ***p < 0.001 and ****p < 0.0001 by unpaired T-test, paired T-test, two-way Anova and two-way Anova with Sidak’s multi comparison test.

After euthanasia we were able to record ECGs of GML isolated hearts placed in a Langendorff perfusion system that maintained an average HR of 232 ± 80 bpm (Fig. 3A). This HR was similar to what we recorded by optical mapping in intact atrial preparations, which included the SAN, the right and left atria (Fig. 3). The atrial preparations of GML were about twice the size of the same preparations in mice of comparable age (24 months old C57BL/6; Figs. 3B and S2). Moreover, while the left atrium of aged mice was slightly smaller than the right atrium, the left atrium of aged GMLs was significantly larger than their right atrium (Fig. S2), suggesting age-related remodeling of atrial chambers. Differences in the size and shape could be noticed also comparing the whole heart in mice and GMLs (Fig. S2C).

We used optical mapping of membrane voltage to record the rate of spontaneous APs and the origin of pacemaker depolarization in the SAN region of GMLs and mice (Fig. 3). This technique also allowed recording the conduction of the electrical impulse from the SAN to the atria in both species (Video S1). We discovered that the average frequency of spontaneous APs generated by the GML SAN was about half of what we recorded in aged mice (24 months old; Fig. 3C,D), but faster than the AP rate reported in aged rats^47^.

Next, we tested the chronotropic response of GML SAN to sympathetic and parasympathetic agonists. As expected, the vagal agonist acetylcholine (50 nM) induced a significant reduction of the SAN AP rate (Fig. 3A,E). On the other hand, the β-adrenergic agonist epinephrine (30 nM) tended to increase the AP rate (Figs. 3A and S3). We then tested saturating dose^29^ of the β-adrenergic agonist isoprenaline (1 µM). Under this condition we observed a significant increase in the generation of spontaneous SAN APs (Fig. 3E). In addition, direct activation of L-type Ca^2+^ channels with the dihydropyridine agonist Bay K 8644 (1 µM), induced a much stronger increase of AP rate in the SANs previously exposed to β-adrenergic stimulation with epinephrine (30 nM) or isoprenaline (1 µM; Fig. 3E). This indicated that direct activation of L-type Ca^2+^ channels in aged GMLs stimulated the chronotropic response more effectively than the global β-adrenergic stimulation. For both adrenergic and vagal agonists, the chronotropic modulation of the atrial preparations was associated with a shift in the origin of pacemaker activity toward the frontal or caudal portion of the SAN (Fig. 3F), in line with our previous observations in mice^50^.

### Pacemaker activity and ionic currents in native GML pacemaker myocytes

**Figure 4:**
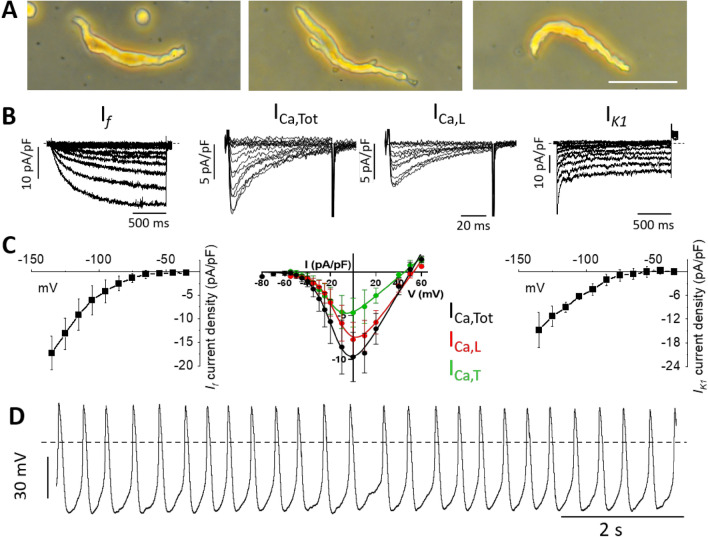
Recordings of ionic currents in SAN myocytes of GML. (**A**) Freshly isolated pacemaker myocytes obtained from the GML SAN tissue (scale bar 50 µm). (**B**) Sample traces of *I*_*f*_, (n = 4, N = 2), total *I*_*Ca*_ (*I*_*Ca,Tot*_) and *I*_*Ca,L*_ (n = 4, N = 2) and *I*_*K1*_ (n = 3, N = 1). (**C**) Current-to-voltage relationships of densities of ionic currents described in panel (**A**) for *I*_*f*_, *I*_*Ca,Tot*_, *I*_*Ca,L*_, *I*_*Ca,T*_ and *I*_*K1*_,*.* (**D**) Action potentials (APs) recorded in SAN pacemaker myocytes from GML under perfusion of Tyrode’s solution.

We then isolated individual pacemaker myocytes from GML SAN to characterize the pacemaker activity at the cellular level (Fig. 4). Immunostaining of pacemaker myocytes within the intact GML SAN region revealed expression of SAN marker f-(HCN4) channel, encoding *I*_*f*_ (Fig. S4), thereby ensuring cell isolation was performed from the pacemaker region.

After enzymatic digestion, we observed single spindle-shaped pacemaker myocytes with self-contractile properties (data not shown), which are similar to those of mice^51^, rats^52^, and humans^19^ (Fig. 4A). Consistent with the expression of HCN4 proteins, we recorded *I*_*f*_ in GML pacemaker myocytes (Fig. 4B) at a density similar to that of mice^53^, i.e. 2.5-fold higher than in human SAN^19^.

In GML SAN myocytes we also recorded *I*_*Ca,L*_ and *I*_*Ca,T*_ (Fig. 4B,C). GML SAN myocytes expressed lower total Ca^2+^ current density (*I*_*Ca,Tot*_ = *I*_*Ca,L*_ + *I*_*Ca,T*_) than murine myocytes, possibly because of the lower *I*_*Ca,T*_. However, *I*_*Ca,L*_ density and its activation threshold were similar to that in the SAN myocytes of mice^26^. Conversely, *I*_*Ca,L*_ peaked at 0 mV, which is more positive than the peak current in mice (− 10 mV)^26^ but similar to that of rats^52^. In addition, in GML SAN myocytes we recorded the inward-rectifier K^+^ current *I*_*K1*_ (Fig. 4B,C) at a density similar to what has been reported in rats^52^.

Current clamp recordings of pacemaker activity in GML SAN myocytes under Tyrode’s solution revealed spontaneous APs with an average beating rate of 148 ± 22 APs/min (n = 7, N = 4; Fig. 4D), consistent with the rate reported in mice and rats^44,47^.

### Correlation between lifetime, HR and weight in primates and other mammals

**Figure 5:**
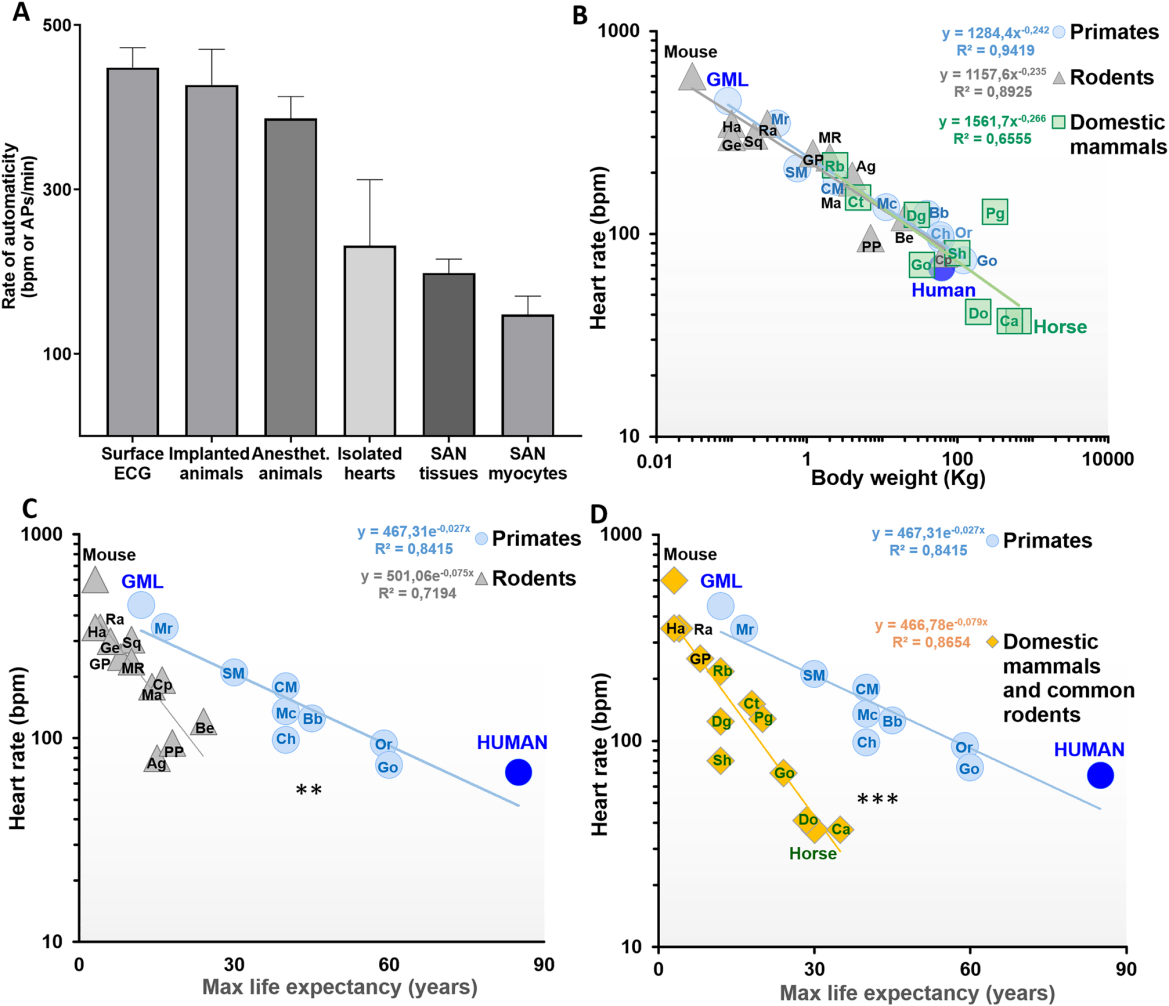
Hierarchical scaling of GML cardiac automaticity and HR versus body weight and life expectancy in primates, rodents and other mammals. (**A**) Decrease of automatic rate at different levels of complexity, going from freely moving animals to single SAN myocytes of GML (n = 13 for surface ECG, n = 4 for implanted animals, n = 6 for anesthetized animals, n = 3 for isolated hearts, n = 16 for SAN tissues and n = 7 for SAN myocytes). (**B**) Correlation between HR and body weight for 10 species of primates, including humans^55^, 12 species of rodents and 9 species of common domestic mammals. Names of the different species are abbreviation as following: Mr = common marmoset, SM = squirrel monkey, CM = capuchin Monkeys, Mc = rhesus macaque, Ch = chimpanzee, Bb = babouin hamadryas, Orang = orangutan, Go = gorilla, Ha = hamster, Ge = Mongolian gerbil, Ra = rat, GP = guinea pig, Sq = north american red squirrel, MR = muskrat, Ma = marmot, Cp = capybara, Ag = agouti, PP = North American Porcupine, Be = north american beaver, Rb = rabbit, Ct = Cat, Sh = sheep, Go = goat, Dg = dog, Pg = pig, Do = donkey and Ca = camel (see methods for each animal reference and scientific name). The correlation for the three distributions is verified by the Spearman analysis (p < 0.001 for primates and rodents, and p < 0.05 for domestic mammals) and the slopes and the intercept of the three logarithmic linear distributions are not significantly different by the covariance (ancova) analysis. (**C**) Correlation between HR and maximal life expectancy for 10 species of primates, including GML and humans^55^, and 12 species of rodents reported in panel (**B**). The correlation for both distributions is verified by the Spearman coefficient (p < 0.001 for primates and rodents) and the slopes of the two semi-logarithmic linear distributions are significantly different by the covariance (ancova) analysis, with p < 0.01. (**D**) Correlation between HR and maximal life expectancy for 10 species of primates, including GML and humans^55^, and 13 species of common domestic mammals reported in panel (**B**). The correlation for both distributions is verified by the Spearman coefficient (p < 0.001 for primates and eutherian mammals) and the slopes of the two semi-logarithmic linear distributions are significantly different by the covariance (ancova) analysis, with p < 0.001.

Comparing different techniques, we observed a decrease in the automatic rate going from the recording of cardiac automaticity in animals’ telemetry and isolated hearts to the analysis of pacemaker activity in atrial preparations and in individual SAN cells (Fig. 5A).

Given the average HR of about 450 bpm that we observed in implanted GMLs and by surface ECG, we estimated that in 12 years of life, the GML’s heart beats about 2.8 billion times. In our colony of GML, 1.6% of all the animals left free to age reached 12 years of age. Similarly, according to the Eurostat sources^54^, in 2019 about 1.5% of the European population was 85 or more years old. Thus, we considered that 12 years in GMLs could be equivalent to 85 years in humans. Given an average HR of 68 bpm^55^, we estimated that a person of 85 years old generated about 3.0 billion heartbeats in a lifetime which is very close to the ~ 2.8 billion heartbeats that we estimated for GML. Conversely, considering an average of 600 bpm in mice^45^ and 350 bpm in rats^47^, and 3 and 4 years of maximal life expectancy for these species, their heart beats ~ 0.9 and ~ 0.7 billion times in their respective life spans. Thus, in a lifetime, humans and GMLs’ hearts seem to be subjected to a similar workload, beating about three times more than mice or rats’ hearts.

To explore these differences, we compared HR, body weight and maximal life expectancy in GMLs, humans and 8 species of primates, 12 of rodents and 9 of other mammal orders. We first observed that primates, rodents and other mammals follow an equivalent regression model for the correlation between HR and body weight (Fig. 5B). Instead, when considering the relation between HR and maximal life expectancy, humans and GMLs can be aligned in the same regression model with other primates, which is significantly less steep than what interpolates the group of rodents or other eutherian terrestrial mammals (Fig. 5C,D). The ratio between the slope indexes of the regression models for rodents (− 0.075) or eutherian terrestrial mammals (− 0.079) versus primates (− 0.027) suggests that at equivalent HR, primates can live about 3 times (2.8–2.9) longer than other mammals.

**Figure 6:**
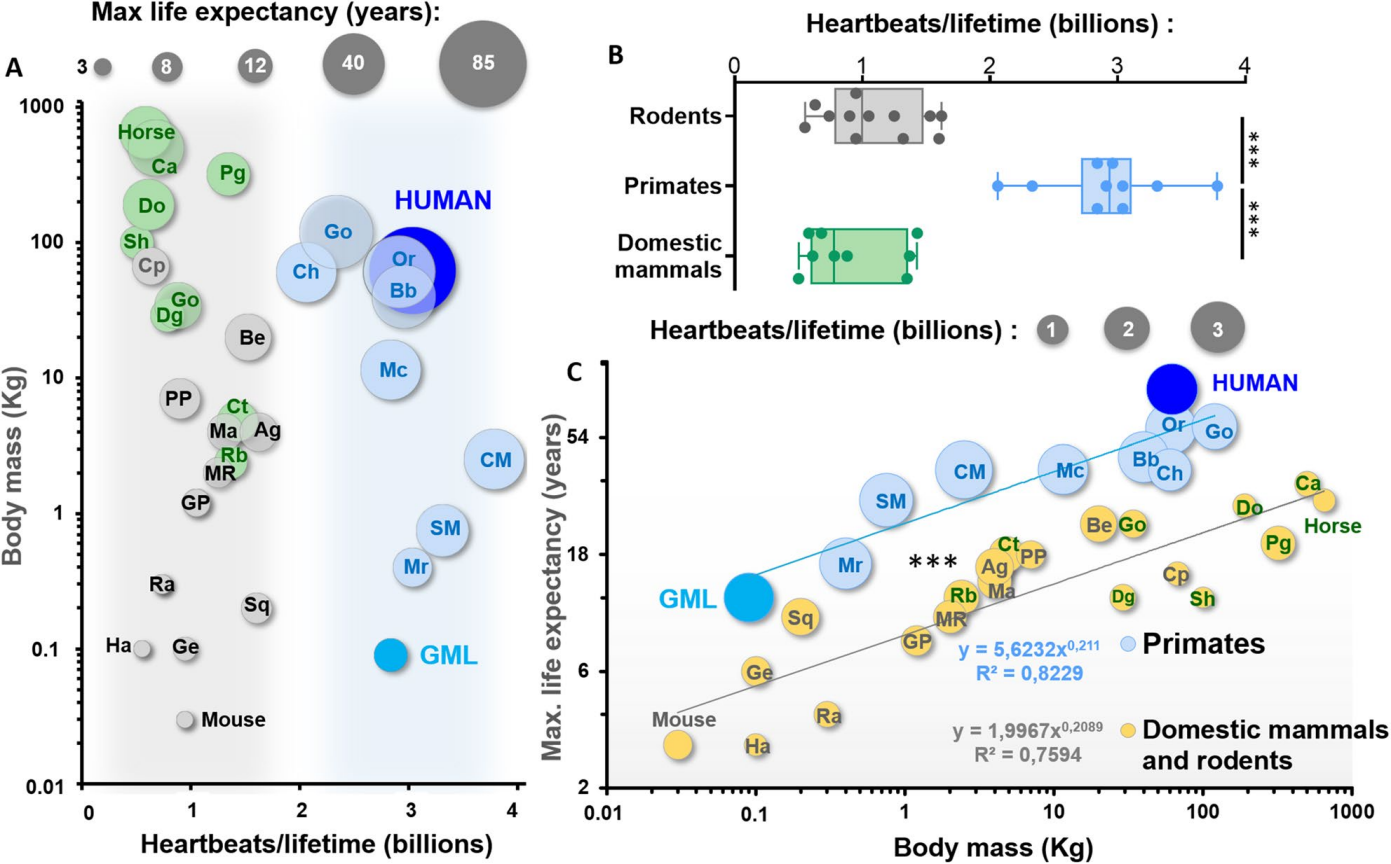
Heartbeats per lifetime in GML, humans, primates and other mammals. (**A**) Relation between Kg of body weight and heartbeats generated in a lifetime for the same animals reported in Fig. 5 (10 primates, 12 rodents and 9 common domestic mammals). Note that primates, including GML and humans, generate about three times more heartbeats than other mammals of similar size. Abbreviations are as in Fig. 5. (**B**) Comparison of heartbeats per lifetime in primates (n = 10), rodents (n = 12) and other mammals (n = 9). ***p < 0.001 by one-way Anova. (**C**) Correlation between maximal life expectancy and body mass in primates and other mammals (n = 10 and n = 21). The correlation for both distributions is verified by the Spearman coefficient (p < 0.001 for primates and eutherian mammals) and the intercept of these two logarithmic linear distributions are significantly different by the covariance (ancova) analysis, with p < 0.001.

These results led us to calculate the number of heartbeats per lifetime in all mammals included in Fig. 5. This comparison showed that most eutherian mammals, including all rodents taken into consideration in our analysis, generate about 1 billion heartbeats in a lifetime^1,5^, independently from body weight (Fig. 6A,B). Conversely, like in humans and GMLs, the heart of other primates generates about 3 billion heartbeats in a lifetime, thus beating three times more than other mammals (Fig. 6A,B). This significant difference was particularly striking when we represented the relation between maximal life expectancy, body mass and heartbeats per lifetime (Fig. 6C). Indeed, this graphic showed that the number of heartbeats per lifetime is equivalent in all primates, including humans and GML, and about three times higher than other mammals. Moreover, it shows that at equivalent body weight, the maximal life expectancy of primates is significantly higher than other mammals.

## Discussion

*Microcebus murinus* or GML (Fig. 1A) is an arboreal and nocturnal lemur among the smallest of the primate order^56^. This lemur belongs to the more ancestral suborder of Strepsirrhini, characterized by less developed brain, placenta and vision^57–59^. Similarly to humans, GML is affected by neurodegenerative diseases as it ages, a feature that is not found in mice^33,60^. Given the importance of the heart–brain crosstalk in HR modulation^37^ and the phylogenetic proximity of *Microcebus murinus* to *Homo sapiens*^31^*,* this lemur could in principle replicate some characteristics of the neural environment that modulates HR in aged people^32–36^. Thus, thanks also to its small size and easy reproduction, GML constitutes a promising model for comparative studies of age-related cardiovascular diseases^56^.

In support of it, we predicted that GMLs, humans and other 8 species of primates generate a comparable number of heartbeats over a lifetime, three times higher than rodents or other eutherian mammals. Thus, the exceptional long-term workload of the heart in primates could cause specific dysfunctions of cardiac activity, different from other mammals commonly used in cardiovascular research. Despite that, to our knowledge, no research has explored the cardiac activity of GML. Here, we used a range of techniques to characterize GML heart automaticity and sinoatrial pacemaker activity, making comparisons with previous data obtained in rodents, humans and other mammals, and investigating the effect of aging on HR.

According to the small size of GML, their HR in awake and anesthetized animals, and their frequency of intrinsic pacemaker activity fall in between those of mice and rats^44,47^. In awake GML we recorded extreme HR as low as 120 and as high as 555 bpm. This highlight a refined autonomic modulation of cardiac activity in GML, possibly related to recurrent short episodes of sleep, occurring in this lemur and similar to what has been highlighted in frugivorous bats to reduce the daily energetic expenditure^42^.

The fast pacemaker activity of GML is sustained by *I*_*f*_ at a density similar to mice^53^ and by *I*_*Ca,L*_ activating at negative voltages, similar to what we described in murine SAN myocytes expressing Ca_v_1.3 channels^26^. Pacemaker activity of GML SAN was regulated by cholinergic and adrenergic agonists (Fig. 3). Autonomic agonists modulated the rate of spontaneous APs and shifted the localization of the pacemaker leading site, although significant β-adrenergic stimulation of pacemaking was observed only at saturating doses of the agonist isoproterenol.

In addition, the GML SAN showed a strong response to direct stimulation of L-type Ca^2+^ channels. Thus, *I*_*Ca,L*_ activation at negative voltages in GML SAN and the positive chronotropic SAN response to the *I*_*Ca,L*_ agonist Bay K 8644 suggest that Ca_v_1.3 channels may play an important role in the pacemaker mechanism of primates*.* Such an hypothesis is consistent with the discovery of inherited channelopathies of the gene expressing Ca_v_1.3, causing SAN dysfunction in humans^21^.

We also recorded Ba^2+^-sensitive *I*_*K1*_ in GML at densities comparable to those observed in rats^52^. *I*_*K1*_ is almost absent in SAN myocytes of large mammals^61^, but it is expressed in mice^62^ and rats^52^. Reasons from the presence or absence of I_K1_ in different mammalian species are unclear. Intriguingly, the apparent reversal potential of Ba^2+^ sensitive I_K1_ in mouse and rat SAN myocytes falls around − 60 mV^52,62^, suggesting GML I_K1_ to be encoded by a channel isoform carrying mixed cationic, rather than K^+^ selective current. Therefore, the maximum diastolic potential in SAN myocytes from the mouse and rat consistently falls around − 60 mV, similar to that recorded in SAN myocytes that do not express this current^52,63^. Thus, the presence of I_K1_ in GML SAN, would not set the maximum diastolic potential close to the K^+^ equilibrium potential as in the ventricle. These observations suggest that expression of I_K1_ in GML, mice and rats could indicate a specificity of small species of mammals.

Taken together, these results suggest that the pacemaker mechanism in the SAN of GML is capable of generating a fast automaticity, necessary to fulfil the high metabolic requirements determined by small size. Accordingly, recorded rates of automaticity in vivo and in intact hearts, isolated SAN tissues and pacemaker myocytes of GML are similar to corresponding rates observed in mice and rats, rodents whose size is comparable to GMLs.

On the other hand, ventricular repolarization of GML (T wave) appeared separated temporally from the depolarization phase by a short ST segment, similarly to what can be observed in large mammals^64^, humans^46,65^ and other small primates^66^, and differently from small rodents^39^. Thus, the longer interval between depolarization and repolarization in GML could be a specificity of primates shared with bigger mammals. As well, enlargement of left atrium that we observed in aged GML but not in mice could constitute a compensatory mechanism similar to the adaptations that ensure adequate blood circulation when the left ventricle does not contract properly in human aged hearts^67^.

In addition, in aged GMLs we observed slowing of basal HR possibly reminiscent of the reduction in maximal HR reported for aged people^12^. Such a result, together with the predicted equivalence of cardiac longevity in GML and humans, suggests the presence of similar age-related cardiac dysfunctions in GML and humans, further supporting the view of GML as a valid model to study heart pathophysiology. A speculative hypothesis could be that evolutionary development of GML cardiac apparatus shares properties with humans and other primates, despite functional divergences originating from its relatively small size, nocturnal life, etc.

Finally, in line with what previously reported in other rodent models of cardiovascular physiology, we noticed a decrease in rates of automaticity from in vivo, to organ, tissue and cellular level. This observation highlights the importance of studying preparation of growing levels of complexity to obtain a complete picture of cardiac activity and its regulation.

After characterizing cardiac activity and the effects of aging on GML, we take advantage of our data in this lemur to compare HR, life expectancy and body weight in a group of 10 primates, including humans, versus rodents or other mammals from different orders. We chose mammals from different orders among domestic species to have reliable data of maximal life expectancy. Thanks to this comparison, we estimated that GMLs, humans and other primates generate three times more heartbeats and have longer life than other eutherian terrestrial mammals with equivalent body weight. Hypothesis could be formulated about a possible antagonistic pleiotropy effect^68^ as a genetic contribution to the exceptional GLM and primates longevity, as described in bats^69,70^. However, detailed genomic analysis would be necessary to investigate such intriguing possibility.

Here we reported that endurance of cardiac pacemaker activity could be an additional important factor to explain the exceptional longevity of primates. These results are in line with the suggested higher longevity of primates versus other mammals^71–73^, which has been in part explained by slower metabolic rate^73^, different fatty acid composition of the plasma membrane^74^, or adaptive advantage determined by large brains^75^. The shared cardiac endurance that we highlighted in primates challenges also the suggested exceptional longevity in humans versus other mammals, based on the privileged conditions of modern societies^1,5,6^. Indeed, our estimations suggest that humans generate approximately the same number of heartbeats per lifetime than other primates, following the same regression laws for the relations between HR, life expectancy and body weight (Figs. 5 and 6). Thus, human’s longevity could just be a primate-derived characteristic, in part exaggerated by the living condition of modern societies.

Further studies will be necessary to establish whether the high number of heartbeats that GMLs, humans and other primates generate in a lifetime are the cause or a consequence of their exceptional longevity^6^. Nevertheless, since cardiac cells cannot regenerate^1^, we can hypothesize that the SAN of primates has to be more resistant that in other mammals and could thus be differently affected by aging. Thus, it is essential to study cardiac pacemaker activity in appropriate models, like GML which, thanks to the equivalent cardiac longevity and phylogenetic proximity with humans^31^, will facilitate the translation of basic research findings into clinical applications against cardiovascular diseases.

## Limitations of the study

Working with primates facilitates the projection of results toward the interpretation of human physiology, but determines scarce access to isolated cardiac preparations particularly in young animals. Our approach was guided by the respect of the “three R’s” of animal welfare. Consequently, we studied isolated cardiac preparations only from animals that have to be euthanized for the decrease in body weight, activity or other vital signs.

On one hand, this approach allowed a general characterization of heart automaticity and sinoatrial pacemaker activity in GML at different levels of preparation complexity, which could be of great help for future studies in cardiology with this species. On the other hand, the use of aged animals complicated the study of β-adrenergic sensitivity of pacemaking in isolated hearts and SAN preparations. Indeed, the mild response to epinephrine that we revealed in these preparations could be due to lower β-adrenergic sensitivity due to age, or inappropriate dose of the agonist used.

A further limitation of the study concerns the comparison of HR, body weight and life expectancy between GML, humans and other species of primates, rodents or mammals of different orders. Our conclusions are indeed based on the average HR reported in literature, in some cases obtained from short-term recordings or from dated articles. However, these data allowed us to obtain estimations that are consistent with previous considerations of life expectancy in primates and propose an interesting and new point of view to explain the exceptional longevity of humans.

## Methods

When possible, we applied the recommendations stated in the ARRIVE guidelines to the manuscript. According to it, all animal procedures were reviewed and conform with the ethical guidelines for the Care and Use of Laboratory Animals published by the US National Institute of Health (NIH Publication No. 85–23, revised 1996) and European directives (2010/63/EU). Male and female *Microcebus murinus*, of 1–12 years of age, were used for telemetry recording with minimally invasive or external electrocardiogram devices that allowed reintegration of the animals to their colony after the experimentation. Conversely, only animals from 5 to 12 years, that after natural aging had to be euthanized for factors hampering normal life, like weight loss, were used for all the other experiments. This approach reduces the need of animals in the respect of the “three R’s” of animal welfare: replacement, reduction, and refinement.

### Housing conditions

All animals were housed in cages equipped with wood branches for climbing activities, as well as wooden sleeping boxes mimicking the natural sleeping sites of the gray mouse lemur (GML). The temperature and the humidity of the rooms were maintained at 25–27 °C and at 55–65%, respectively. In captivity, the artificial daily light–dark cycles within the housing rooms are changed to mimic season alternation, with periods of 6 months of summer-like long days (14 h of light and 10 h of darkness, denoted 14:10) and 6 months of winter-like short days (10 h of light and 14 h of darkness, denoted 10:14). Animals were fed ad libitum with fresh fruit, worms and a homemade mixture as in Hozer et al.^76^.

GMLs used for the implantation of electrocardiogram transmitters were hosted at the GML colony of Brunoy (MECADEV, MNHN/CNRS, IBISA Platform, agreement F 91.114.1, DDPP, Essonne, France) under the approval of the Cuvier Ethical Committee (Committee number 68 of the “Comité National de Réflexion Éthique sur l’Expérimentation Animale”), under authorization number 68-018. In the respect of the principle of “three R’s” of animal welfare, the use of these data, collected during another project for other purposes, allowed reduction of animal use since we did not have to implant new animals for the present project. All the other GMLs came from the colony housed at RAM-CECEMA (license approval 34-05-026-FS, DDPP, Hérault, France). Their use was conducted under the approval of the CEEA-LR Ethical Committee (Committee number 36, 34323-2021121308124262). Animals were euthanized at the end of their lives and all organs were collected immediately for biomedical research.

Electrocardiogram recordings.

One-lead electrocardiograms were recorded from freely moving and anaesthetized GMLs. For electrocardiogram recordings in freely moving animals implanted with a transmitter, surgery was conducted in sterile conditions, under veterinary supervision. After administration of diazepam (Valium, 1 mg/100 g, i.m.) and buprenorphine (0.005 mg/100 g, i.m.), anesthesia was induced and maintained by 1–3% isoflurane inhalation. Body temperature was maintained with a heating pad, and the animal’s eyes were protected with ocular gel (Ocry − gel; Laboratoire TVM, Lempdes, France). A small transmitter (PhysioTel F20-EET, 3.9 g, 1.9 cc; Data Sciences International TM, DSI, St. Paul, United States) connected with 1 pair of electrode wires (silicon elastomer insulated stainless-steel wires, diameter: 0.3 mm) was inserted inside the peritoneal cavity of the animal. Electrode wires were led subcutaneously from the abdomen to the thorax muscle and were sutured using non-absorbable polyamide monofilament suture thread, similarly to the procedure described in mice^44^. After surgery, nociception was minimized by subcutaneous injection of analgesic and anti-inflammatory drugs (meloxicam, 0.2 mg/100 g). Electrocardiogram signals were recorded using a telemetry receiver and an analog-to-digital conversion data acquisition system (Data Sciences International TM, DSI, St. Paul, United States). HR and HRV data were analyzed with the dedicated software Labchart 8 (ADInstruments, Dunedin, New Zealand) and ecgAUTO v3.3.5.12 (emka TECHNOLOGIES, Paris, France).

For external electrocardiograms, we used solid gel electrodes and the uECG Holter of Ultimate Robotics (Kyiv, Ukraine), to obtain one lead recording of HR in GML males of different ages. A homemade vest helps to keep the uECG Holter on the GML back, ensuring that the electrode touches the skin of the animals at the level of the shoulders and pelvis. Before that, the animals were shaved in the contact points with the gel electrodes, to facilitate the conduction of the signal through the skin. Electrocardiogram recordings were obtained through dedicated application software provided by Ultimate Robotics and analyzed with the dedicated software Labchart 8 (ADInstruments, Dunedin, New Zealand). 5 min of electrocardiogram recordings obtained after 90 min of adaptation to the ECG vest in the normal environment of GML were used to obtain resting HR.

For electrocardiogram recordings under anesthesia, we constantly exposed GML to 1.5% isoflurane. GML Body temperature was continuously maintained at 36–37 °C using a heated pad connected to a controller that received feedback from a temperature sensor attached to the animal. Ag/AgCl gel-coated electrocardiogram electrodes (Unomedical; Herlev, Danimarca) were attached to the superior right and to the two inferior limbs of GML. The electrodes were connected to a standard one-lead electrocardiogram amplifier module (EMKA Technologies, Paris, France), which included high- and low-pass filters (set to 0.05 Hz and 500 Hz, respectively) and a gain selection device (set to 1000-fold). Signals were digitized continuously at 2 kHz and recorded using an IOX data acquisition system (emka TECHNOLOGIES, Paris, France). The recordings were carried out for a 45-min period, and the software ECGAuto (emka TECHNOLOGIES, Paris, France) was used to perform offline analysis of the recorded data. For each GML the mean HR, its standard deviation and the parameters characterizing the electrocardiogram wave, were calculated at 30-min intervals starting 10 min after the beginning of each 45-min recording.

### Langendorff perfused hearts

After general anesthesia, consisting of 0.1 mg/g of ketamine (Imalgène, Merial, Bourgelat France) and complete loss of hind-limb reflex or any other body sensation we quickly isolated the heart from the GML thoracic cage (1–2 min for heart isolation), and we cannulated it to insure a minimal perfusion with preoxygenated Tyrode’s solution during transportation to the laboratory. GML heart was transported to the laboratory for experimentation within 5–10 min after isolation, and quickly mounted on a Langendorff apparatus (Isolated heart system; emka TECHNOLOGIES, Paris, France) at a constant pressure of 80 mm Hg with normal Tyrode’s solution. Perfused hearts were immersed in the water-jacked bath and maintained at 36 °C. Electrocardiograms were continuously recorded by Ag–AgCl electrodes positioned on the epicardial side of the right atrium close to the SAN and near the apex. HR was allowed to stabilize for at least 30 min before perfusion of epinephrine (30 nM) or acetylcholine (50 nM).

### Atrial preparation and optical voltage mapping

Atrial preparations (including the SAN and the right and left atria) were obtained as described previously^29^. Briefly, after general anesthesia with ketamine and complete loss of hind-limb reflex (see above), we removed the heart from the thoracic cage of the animal. Then, we cut the coronary sinus and, starting from it, we removed the ventricles from the heart. We used a stereomicroscope (SZX16, Olympus; Tokyo, Japan) with low magnification (7×) to trans-illuminate and directly visualize the remaining atrial preparation. We identified the SAN region using the borders of the superior and inferior vena cava, the crista terminalis and the interatrial septum as landmarks^77^. The atrial preparation was pinned to the bottom of an optical chamber (Fluorodish, FD35PDL-100, WPI; Sarasota, FL) coated with ~ 2 mm of clear Sylgard (Sylgard 184 Silicone elastomer kit; Dow Corning; Midland, MI). To avoid interference from secondary pacemaker tissues we removed the atrioventricular node from the preparation.

For comparative experiments, 24-month-old mice (C57BL/6) were used to match the old age of *Microcebus murinus* (from 6 to 11 years old) from which we obtain tissue preparation of the SAN. As for *Microcebus murinus* the investigation with mice conforms to the European directives (2010/63/EU) for the Care and Use of Laboratory Animals and was approved by the French Ministry of Agriculture (N° D34-172-13). Briefly, mice were anaesthetized with 0.01 mg/g xylazine (Rompun 2%, Bayer AG, Leverkusen Germany), 0.1 mg/g ketamine (Imalgène, Merial, Bourgelat France) and 0.2 mg/g Na-Pentobarbital (CEVA, France). Then, after complete loss of hind-limb reflex or any other sensation, we removed the heart from the thoracic cage of the animal and we further dissected it to obtain the entire atrial preparation including the SAN and the atria^29^.

To analyze voltage changes in the SAN preparation we loaded it by immersing the tissue in a Tyrode’s solution containing the voltage-sensitive indicator Di-4-ANEPPS (2 µmol/L; AAT Bioquest, Sunnyvale, California). This immersion was performed at room temperature (20–22 °C) and lasted for at least 30 min. To maintain proper oxygenation, the chamber containing the tissue was maintained under agitation for the whole loading period. After the loading step, the tissue was washed in dye-free Tyrode’s solution for 15 min. During this step, we slowly increase the temperature to 34–36 °C. The atrial preparation was then constantly superfused at 34–36 °C and imaged by high-speed optical voltage mapping (1000–333 frames/s) on a MiCAM03 Camera—256 × 256 pixel CMOS sensor, 17.6 × 17.6 mm (SciMedia; Costa Mesa, CA). This camera was mounted on a THT microscope, with two objectives (PLANAPO 2X Leica as magnification lens and PLANAPO 1.6X Leica as eyepiece lens) that generated a field of view of 22 × 22 mm. A system constituted by a 150 W halogen light and a built-in shutter (SciMedia; Costa Mesa, California) was used as the excitation source of light for the voltage dye. The filter set included a 531/50 nm excitation filter, 580 nm dichroic mirror, and 580 long-pass emission filter. To avoid motion artefacts, we blocked mechanical activity using blebbistatin (10 µM; Tocris Bioscience; Bristol, UK)^29^. Optical raw data were analysed using dedicated software from the camera developer, BV workbench (Brainvision; Tokyo, Japan), in combination with ClampFit (ver. 10.0.7, Molecular Devices, LLC; San Jose, California).

### Isolation of SAN myocytes

The GML SAN tissue was isolated as described above (see paragraphs above) and cut in tissue strips immediately after transport to the laboratory for experimentation. Strips were then transferred into a low-Ca^2+^, low-Mg^2+^ solution containing (in mM): 140.0 NaCl, 5.4 KCl, 0.5 MgCl_2_, 0.2 CaCl_2_, 1.2 KH_2_PO_4_, 50.0 taurine, 5.5 d-glucose and 1.0 mg/ml BSA, plus 5.0 mM HEPES–NaOH (adjusted to pH 6.9 with NaOH). The tissue was enzymatically digested by adding 229 U/ml collagenase type II (Worthington Biochemical Corporation), 1.9 U/ml elastase (Boehringer Mannheim), 0.9 U/ml protease (Sigma-Aldrich), 1 mg/ml BSA, and 200 μM CaCl_2_. Tissue digestion was performed for a variable time of 9–13 min at 35 °C with manual agitation using a flame-forged Pasteur pipette. Tissue strips were then washed and transferred into a medium containing (in mM): 70.0 l-glutamic acid, 20.0 KCl, 80.0 KOH, 10.0 (±) d-β-OH-butyric acid, 10.0 KH_2_PO_4_, 10.0 taurine, 1 mg/ml BSA, and 10.0 HEPES–KOH, adjusted at pH 7.4 with KOH. SAN myocytes were manually dissociated in KB solution at 35 °C for about 10 min. Cellular automaticity was recovered by readapting the myocytes to physiological extracellular Na^+^ and Ca^2+^ concentrations by adding first a solution containing (in mM): 10.0 NaCl, 1.8 CaCl_2_, and subsequently normal Tyrode’s solution containing 1 mg/ml BSA. The final storage solution contained (in mM): 100.0 NaCl, 35.0 KCl, 1.3 CaCl_2_, 0.7 MgCl_2_, 14.0 l-glutamic acid, 2.0 (±)d-β-OH-butyric acid, 2.0 KH_2_PO_4_, 2.0 taurine, and 1.0 mg/ml BSA, pH 7.4. Cells were then stored at room temperature until use. All chemicals were obtained from Sigma-Aldrich, except for the (±)d-β-OHbutyric acid, which was purchased from Fluka Chemika. For electrophysiological recording, SAN myocytes in the storage solution were harvested in special custom-made recording Plexiglas chambers.

### Patch-clamp recordings of *Microcebus murinus* SAN myocytes

Myocytes isolated from the *Microcebus murinus* SANs were prepared as described in the previous sections. The basal extracellular Tyrode’s solution used in all recordings contained (in mM): 140.0 NaCl, 5.4 KCl, 1.8 CaCl2, 1.0 MgC_l2_, 5.0 HEPES–NaOH, 5.5 and d-glucose (adjusted to pH 7.4 with NaOH). To measure the hyperpolarization-activated current *I*_*f*_, we employed standard Tyrode’s solution in the presence or absence of 2 mM Ba^2+^ to block the inward rectifier K^+^ current *I*_*K1*_, without affecting *I*_*f*_^78^. *I*_*K1*_ was subtracted from *I*_*f*_ as the net Ba^2+^-sensitive conductance, while *I*_*f*_ was extracted as the currents not blocked under perfusion of Ba^2+^, but subsequently inhibited in the presence of 5 mM Cs^+^. *I*_*Ca,*L_ and *I*_*Ca,T*_ were recorded as previously described^26^. In particular, I_CaToT_ (I_CaT_ + I_CaL_) was recorded from a holding potential of − 80 mV in isolated SAN myocytes. Then after switching HP to − 55 mV we inactivated I_CaT_ to obtain only I_Ca,L_.

AP firing activity was measured in the current clamp configuration. Patch-clamp electrodes had a resistance of 4–5 MΩ when filled with an intracellular solution containing (in mM): 130.0 K^+^-aspartate; 10.0 NaCl; 2.0 ATP-Na^+^ salt, 6.6 creatine phosphate, 0.1 GTP-Mg^2+^, 0.04 CaCl_2_ (pCa = 7.0), and 10.0 Hepes KOH (adjusted to pH 7.2 with KOH). Pacemaker activity of SANs and PML cells were recorded under perforated patch conditions by adding β-escin (50 μM) to the pipette solution.

### Immunofluorescence analysis

Immunofluorescence analysis was performed on SAN tissue as in the reference^79^. Tissues were fixed in 4% paraformaldehyde for 6 h. Then these samples were permeabilized for 30 s in − 20 °C acetone, preincubated for 30 min with 0.5% BSA in PBS. SAN tissues were then incubated for 1 h at 37 °C with primary antibodies against HCN4, (1:100, Alomone), sarcomeric alpha-actinin, clone EA-53 (1:100, Sigma-Aldrich). Specific staining was revealed using anti-mouse or anti-rabbit Alexa Fluor 488 and Alexa Fluor 555-conjugated secondary antibodies (InVitrogen) with Hoechst33358 nuclear stain (0.1 µg/ml) for 30 min at 37 °C.

### Statistical analysis

HR, maximal life expectancy, body size and the resulting number of heartbeats per lifetime were obtained from data available in the literature for different mammals including 10 species of primates, 12 species of rodents and 9 species of domestic mammals from different orders. In particular we took in consideration the species GML, common marmoset (*Callithrix jacchus*)^80,81^, squirrel monkey (*Saimiri sciureus*)^82,83^, capuchin Monkeys (*Cebus apella*)^66^, rhesus macaque (*Macaca mulatta*)^84,85^, chimpanzee (*Pan troglodytes schweinfurthii*)^86^, Babouin hamadryas (*Papio hamadryas*)^87^, orangutan (*Pongo pygmaeus pygmaeus*)^88^ and gorilla (*Gorilla gorilla gorilla*)^89^, in comparison to humans^55^ for the primate order; mouse^45^, hamster^64,90^, rat^47^, Mongolian gerbil (*Meriones unguiculatus*)^91,92^ guinea pig^93^, red North American red squirrel (*Tamiasciurus hudsonicus*)^2^, muskrat (*Ondatra zibethicus*)^94^, marmot (*Marmota monax*)^95^, capybara (*Hydrochoerus hydrochaeris*)^96,97^, agouti (*Dasyprocta primnolopha*)^98^, North American Porcupine (*Erethizon dorsatum*)^95^, north american beaver (*Castor canadensis*)^94^ for rodents and rabbit^99^, dog^99,100^, sheep^101^, cat^102^, pig^103^, goat^104,105^, donkey^106^, horse^107^ and camel^108^ for the domestic mammals. HR were reported from results obtained in unanesthetized and mainly freely moving animals under resting conditions. Significance was evaluated through paired or unpaired Student’s T test, one-way- and two-way ANOVA and comparison between regression law as specified in figure legends. When testing statistical differences, results were considered significant with p < 0.05. Data analysis was performed with GraphPad Prism 9.0 and IBM SPSS Statistics 28.0.0.0.

## Supplementary Information

Supplementary Video 1.

Supplementary Information 1.

## Data Availability

The datasets generated and/or analyzed during the current study are not publicly available due to the novelty of such research, but are available from the corresponding author on reasonable request.
