# Supplementary material for: Characterization of sinoatrial automaticity in Microcebus murinus to study the effect of aging on cardiac activity and the correlation with longevity
Source: Sci Rep. 2023 Feb 21;13:3054. doi: 10.1038/s41598-023-29723-5 (PMC9944915; doi:10.1038/s41598-023-29723-5)
Supplement: Supplementary file 2 — Supplementary Information 1. [file 41598_2023_29723_MOESM2_ESM.docx]

**Supplementary Information for:**

**Characterization of sinoatrial automaticity in *Microcebus murinus* to study the effect of aging on cardiac activity and the correlation with longevity.**

Mattia L. DiFrancesco^1,2,*^, Manon Marrot^1,2^, Eleonora Torre^1,2^, Pietro Mesirca^1,2^, Romain Davaze^3^, Corinne Lautier^4^, Pascaline Fontes^4^, Joël Cuoq^4^, Anne Fernandez^3^, Ned Lamb^3^, Fabien Pifferi^5^, Nadine Mestre-Francés^4,6^, Matteo E. Mangoni^1,2^ and Angelo G. Torrente^1,2^.

^1^Institut de Génomique Fonctionnelle, Université de Montpellier, CNRS, INSERM, Montpellier, France.

^2^LabEx Ion Channels Science and Therapeutics (ICST), Sophia Antipolis, France.

^3^Institute of Human Genetics, CNRS and University of Montpellier, Montpellier, France.

^4^MMDN Univ Montpellier, EPHE, INSERM, Montpellier, France.

^5^UMR CNRS/MNHN 7179, Mécanismes Adaptatifs et Evolution, Brunoy, France

^6^ PSL Research University, Paris, France

*Present address: IRCCS Ospedale Policlinico San Martino, Largo Rosanna Benzi 10, 16132, Genoa, Italy.

Corresponding authors:

**Drs. Angelo G. Torrente and Matteo E. Mangoni**

Institut de Génomique Fonctionnelle, Département de Physiologie et Cancer,

CNRS UMR 5203, INSERM U 1191, Université de Montpellier

34097 Montpellier, France

tel. +33 (0)4 34 35 92 46

*e-mail*: [matteo.mangoni@igf.cnrs.fr](mailto:matteo.mangoni@igf.cnrs.fr) and [angelo.torrente@igf.cnrs.fr](mailto:angelo.torrente@igf.cnrs.fr)

**Supplementary materials**.

Supplemental figures:


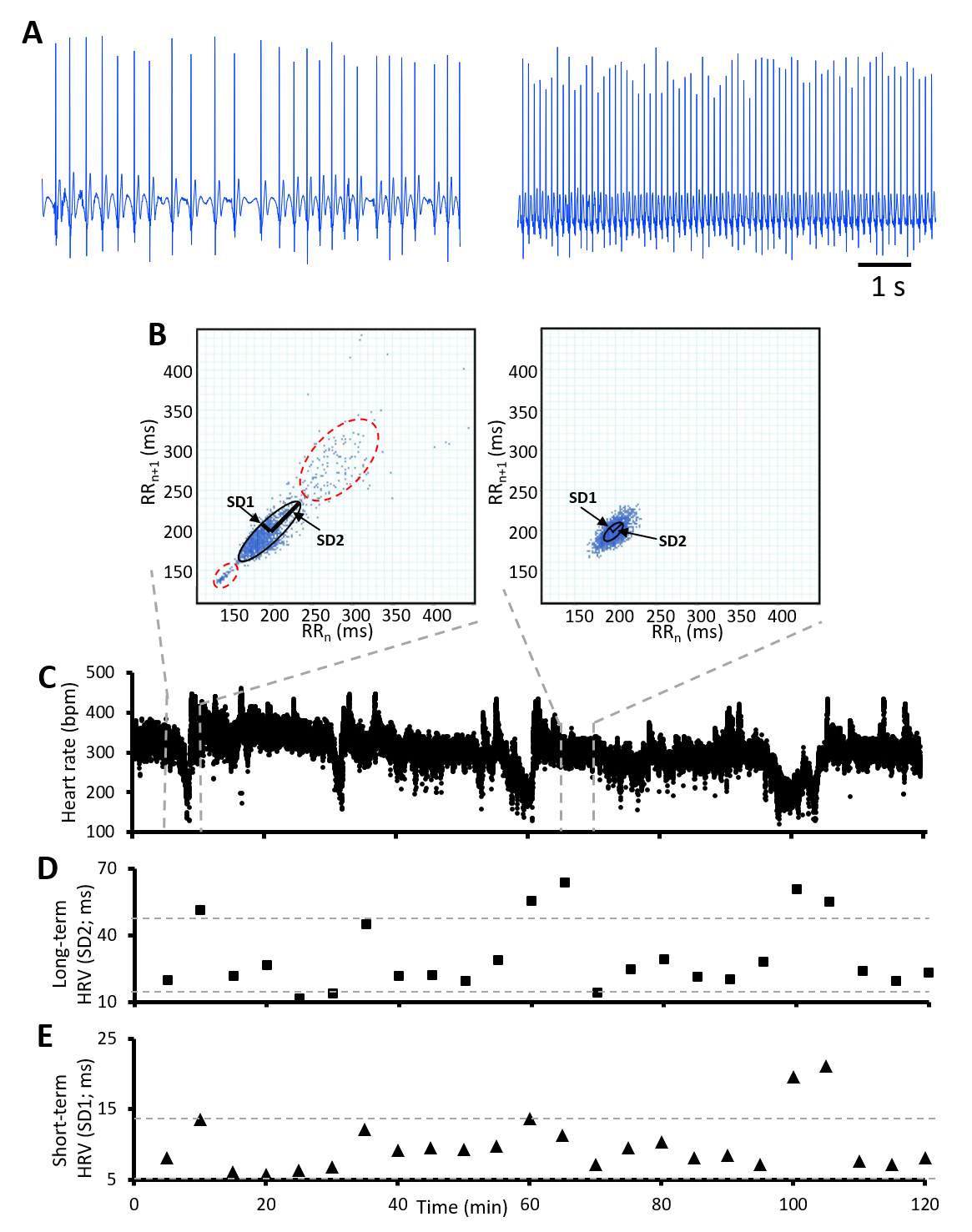


**Figure S1: Indexes of long- and short-term HRV in freely moving GMLs.**

**A**: ECG recordings showing very long and very short RR intervals. **B:** Poincaré plots obtained analyzing intervals of 5 min during periods of high (left panel) and low (right panel) HRV in D. In both panels note the standard deviations from axis 1 and 2 (SD1 and SD2, respectively) which measure short- and long -term HRV, respectively. In the left panel note the presence of two clouds of points (red dotted ellipses) separated from the main one (black ellipse). The points belonging to the red dotted ellipse in the upper part of the panel correspond to slow HR (long RR intervals), while those belonging to the red dotted ellipse in the lower part of the panel correspond to very fast HR (short RR intervals). Such subdivision is absent in the right panel, which represents by Poincaré an interval of 5 min with homogeneous HR. **C:** HR versus time for a period of 120 min. **D** and **E:** Plots obtained by measuring the average long- and short-term HRV (SD2 and SD1, respectively) for intervals of 5 minutes of the recording in D. Dashed gray lines in E and F indicate the threshold of mean ± SD of the reported parameters in this panel.


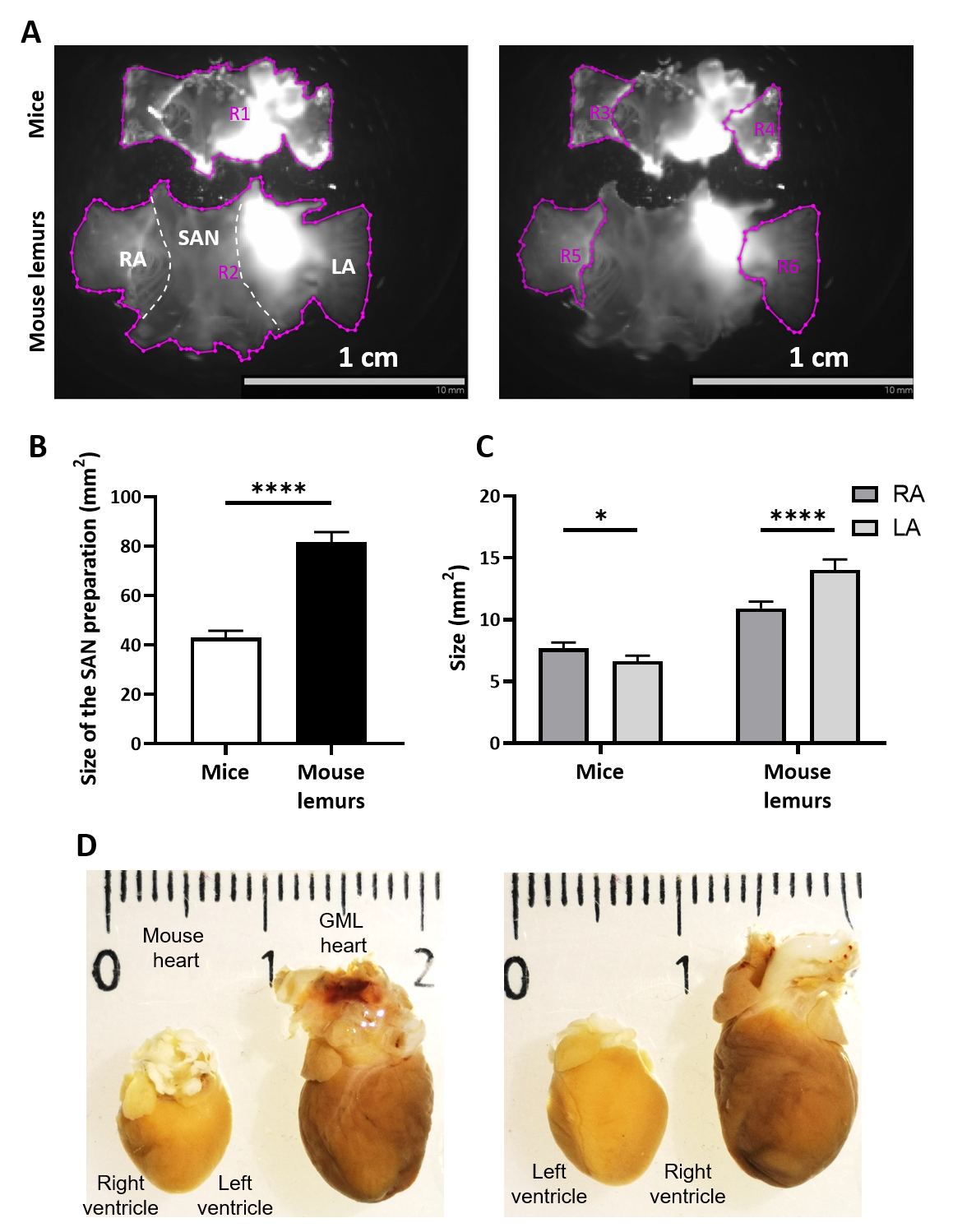


**Fig. S2: Comparative analysis of cardiac preparations of GMLs** **and mice.**

**A:** Atrial preparations of mice and GMLs including SAN, right and left atria (RA and LA, respectively). Purple lines indicate the surface of the regions including the whole SAN preparation (R1 and R2 respectively), the surface of RA (R3 and R4, respectively) and the surface of LA in mice and GML (R5 and R6, respectively). **B:** Size of the whole atrial preparations of mice and GML represented in A. **C:** Size of RA and LA. N mice = 14 and n GMLs = 11. **D:** Examples of whole hearts in mice and GML. *p<0.05, ****p<0.0001 by unpaired T-test and One-way Anova with Sidak’s multi-comparison test.

**
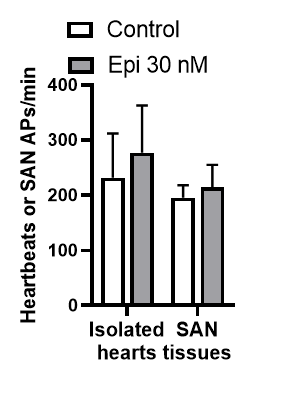
**

**Fig. S3: β-adrenergic response to Epinephrine:**

HR changes (heartbeats or APs/min), before and after Epinephrine in Langendorff perfused hearts (n=3) or atrial preparation of GML (n=9).

**
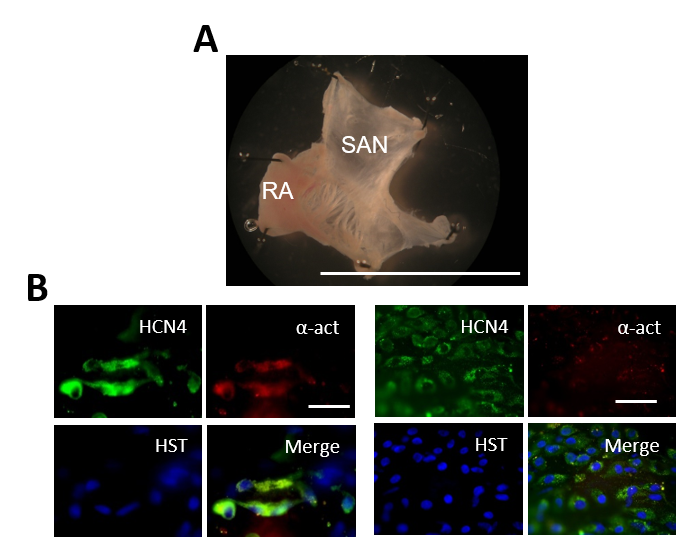
**

**Fig. S4: Immuno-detection of cardiac-pacemaker markers from GML SAN tissue and isolated myocytes.**

**A:** Dissection of the atrial preparation used to isolate SAN myocytes, showing the SAN and the right atrium (scale bar = 10 mm). **B:** Whole-mount imaging from different angles of sectioning of pacemaker myocytes within the intact SAN tissue stained for HCN4, α-actinin and nuclei (Hoechst33358; HST); scale bar = 30 µm (left panel) and = 40 µm (right panel).

Supplemental videos:

**Video S1: Dynamics of pacemaker generation and conduction within the murine (up) and GML (down) atrial preparation** (Right atrium on the left, left atrium on the right and SAN in the center)**.** Green to red colors indicated the intensity of depolarization in arbitrary units of fluorescence.
